# Gemcitabine as a molecular targeting agent that blocks the Akt cascade in platinum-resistant ovarian cancer

**DOI:** 10.1186/1757-2215-7-38

**Published:** 2014-04-09

**Authors:** Hiroshi Kawaguchi, Yoshito Terai, Akiko Tanabe, Hiroshi Sasaki, Masaaki Takai, Satoe Fujiwara, Keisuke Ashihara, Yoshimichi Tanaka, Tomohito Tanaka, Satoshi Tsunetoh, Masanori Kanemura, Masahide Ohmichi

**Affiliations:** 1 Department of Obstetrics and Gynecology, Osaka Medical College, 2-7, Daigaku-machi, Takatsuki, Osaka 569-8686, Japan

**Keywords:** Gemcitabine, Ovarian cancer, Platinum resistance, PI3K/Akt cascade, Apoptosis, VEGF

## Abstract

**Background:**

Gemcitabine (2′, 2′ –difluorodeoxycytidine) is one of many nonplatinum drugs that exhibit activity in recurrent, platinum-resistant ovarian cancer. However, the molecular mechanisms by which Gemcitabine treatment inhibits the proliferation of platinum-resistant ovarian cancer cells still remain unclear. We investigated whether Gemcitabine increases the efficacy of Cisplatin in platinum-resistant ovarian cancer models *in vitro* and *in vivo*.

**Methods:**

We used Cisplatin-resistant Caov-3 cells, A2780CP cells and Cisplatin-sensitive A2780 cells to examine the sensitivity of the cell viability of Cisplatin and Gemcitabine using a 3-(4,5-dimethylthiazol-2-yl)-5-(3-carboxymethoxyphenyl)-2-(4-sulfophenyl)-2H-tetrazolium (MTS) assay and the sensitivity of the invasive activity of Cisplatin and Gemcitabine using an invasion assay with Matrigel. We examined the Akt kinase activity and matrix metalloproteinase 9 (MMP9) expression following Cisplatin and Gemcitabine treatment using a Western blot analysis and the mRNA expression of vascular endothelial growth factor (VEGF) using semi-quantitative RT-PCR. Moreover, we evaluated the effects of Cisplatin and Gemcitabine on the intra-abdominal dissemination of ovarian cancer *in vivo*.

**Results:**

Gemcitabine significantly inhibited Cisplatin-induced Akt activation in the Caov-3 and A2780CP cells, but not in the A2780 cells. In the presence of Gemcitabine, Cisplatin-induced growth inhibition and apoptosis were significantly enhanced in the Caov-3 and A2780CP cells. Co-treatment with Cisplatin and Gemcitabine almost completely inhibited invasion of both types of cells through the Matrigel; however, neither Cisplatin nor Gemcitabine alone inhibited the invasion of both types of cells. Gemcitabine inhibited not only the Cisplatin-induced activation of Akt, but also the MMP9 and mRNA expression of VEGF. Moreover, treatment with Gemcitabine increased the efficacy of Cisplatin-induced growth inhibition of the intra-abdominal dissemination and production of ascites in the athymic nude mice inoculated with Caov-3 cells.

**Conclusions:**

We herein demonstrated that Gemcitabine inhibits the Akt kinase activity and angiogenetic activity following treatment with Cisplatin in platinum-resistant ovarian cancer cells. These results provide a rationale for using Gemcitabine in clinical regimens containing molecular targeting agents against platinum-resistant ovarian cancers.

## Introduction

Ovarian cancer is a major cause of death among patients with gynecological malignancies. There was some improvement in the survival time following the introduction of platinum (Cisplatin or Carboplatin) and Paclitaxel therapy; however, the likelihood of success in the treatment of females with advanced, recurrent or persistent ovarian cancer has remained largely unchanged for four decades [1]. Therefore, there is a need to consider the use of second-line chemotherapeutic options in ovarian cancer patients [2-8]. However, the patient response rates to second-line therapy are strikingly different depending on the platinum sensitivity of the cancer. Furthermore, clear cell carcinoma and mucinous adenocarcinoma in advanced stages (III and IV) have been reported to be associated with a lower survival rate due to resistance to platinum-based chemotherapy [9-11]. Accordingly, the important determinant of the patient prognosis thus seems to be whether the ovarian cancer is sensitive or resistant to platinum.

The balance between cellular survival and apoptosis determines the sensitivity of cells to chemotherapeutic drug-induced apoptosis. Therefore, it is possible that antiapoptotic signals, such as the phosphatidylinositol 3-kinase (PI3K)-Akt survival cascade, plays a role in tumor sensitivity to chemotherapeutic drugs. We previously reported that Akt inactivation sensitizes human ovarian cancer cells to Cisplatin [12,13] and Paclitaxel [14]. Hence, the inhibition of antiapoptotic signals, such as those mediated by the Akt pathway, has been proposed to be a promising strategy for enhancing the efficacy of conventional chemotherapeutic agents [15]. Since the PI3/Akt cascade is involved in Cisplatin resistance, inhibiting this cascade using gene transfection is effective in reversing Cisplatin resistance [12,16]. However, small molecular agents that block the Akt cascade have not so far been available in the clinical setting.

Gemcitabine (2′, 2′ –difluorodeoxycytidine) is one of many non-platinum drugs that exhibit activity in recurrent, platinum-resistant ovarian cancer [17]. Interestingly, preclinical studies have suggested that Gemcitabine may have an additive or synergistic effect when combined with Cisplatin [18]; clinical studies of groups of females who have previously received multiple lines of therapy support this notion [19,20]. Moreover, it has been reported that Gemcitabine induces apoptosis in human pancreatic cancer cells, in part, by downregulating the PI3K-Akt signaling pathway [21].

These considerations led us to examine whether Gemcitabine inhibits the PI3K/Akt signaling pathway in ovarian cancer cells. In the present study, we found that Gemcitabine attenuates the PI3K/Akt cascade and increases the efficacy of Cisplatin in Cisplatin-resistant ovarian cancer cell lines both *in vitro* and *in vivo*.

## Materials and methods

### Reagents/antibodies

Gemcitabine hydrochloride was purchased from LKT Laboratories, Inc. (Minnesota, USA) and dissolved in sterile water. Cisplatin was purchased from Sigma-Aldrich (Dorset, UK). Antiphospho-Akt (ser473), anti-Akt, anti-PARP, anti-MMP9 antibodies were purchased from Cell Signaling Technology (Beverly, MA).

### Cell lines

The human ovarian cisplatin-resistant Caov-3 cells, cisplatin-resistant A2780CP cells and cisplatin-sensitive A2780 cells were obtained from the American Type Culture Collection (Rockville, MD). The cells were cultured at 37°C/5% CO_2_ in DMEM supplemented with 10% FBS in a humidified atmosphere. All mice were purchased from Japan SLC (Shizuoka, Japan).

### Proliferation assay

Cell proliferation was assessed using an 3-(4,5-dimethylthiazol-2-yl)-5-(3-carboxymethoxyphenyl)-2-(4-sulfophenyl)-2H-tetrazolium (MTS) assay. We examined the effects of Cisplatin or Gemcitabine alone and in combination on the proliferation of the Caov-3, A2780CP and A2780 cells using a proliferation assay.

A total of 5 × 10^5^ cells cultured at 37°C in a humidified, 5% CO_2_ atmosphere in serum-free DMEM for 24 hours and pretreated with PBS or Cisplatin at various concentrations or 100 nM of Gemcitabine for 12 hours followed by PBS or Cisplatin at various concentrations were seeded into each well. The assays were performed after 24 hours by adding 20 μl/well of the CellTiter 96® AQueous One Solution Reagent (Promega Corporation, USA) directly to the culture wells, incubating for one to two hours and then recording the absorbance at 490 nm with a 96-well plate reader. All experiments were carried out in quadruplicate, and the viability was expressed as the ratio of the number of viable cells treated with Cisplatin to the number of viable cells treated without Cisplatin. Each point represents the mean ± SD of four experiments. We examined whether the co-treatment with cisplatin and gemcitabine leads to synergistic inhibition of cell viability in the Caov-3, A2780CP and A2780 cells, and calculated synergistic effects using a Mixlow method of Assessing Drug Synergism/Antagonism [22]. The MixLow program was coded in the R (R Foundation for Statistical Computing, Austria).

### Cell invasion assay

The invasive potential was assessed using the Invasion assay. We examined the effects of Cisplatin or Gemcitabine alone and in combination on the invasive potential of the Caov-3, A2780CP and A2780 cells using an invasion assay. A total of 5 × 10^5^ cells cultured at 37°C in a humidified, 5% CO^2^ atmosphere in serum-free DMEM for 24 hours and pretreated with PBS or 100 μM of Cisplatin or 100 nM of Gemcitabine for 12 hours followed by PBS or 100 μM of Cisplatin were seeded into the upper wells coated with a thin layer of Matrigel. The lower chamber was coated with 600 μL of DMEM. Following 24 hours of incubation at 37°C, the non-invading cells on the surface of the Matrigel-coated membrane were removed by scraping with a cotton swab. Cells that had migrated through the Matrigel were stained with hematoxylin. Following several washes with PBS, the stained cells were manually counted for three independent experiments. Each point represents the mean ± SD of four experiments.

### Western blot analysis

The cells were starved and treated with PBS or 100 μM of Cisplatin for 24 hours with or without being pretreated with 100 nM of Gemcitabine for 12 hours. The cells were then washed twice with ice-cold phosphate-buffered saline, lysed and separated to cytoplasmic and nuclear fractions using the Nuclear Extract Kit according to the manufacturer’s protocol (Active Motif, Carlsbad, CA). To detect Akt, phosphorylated Akt, MMP9 and PARP proteins, we separated the proteins using SDS polyacrylamide gel electrophoresis and electrotransferred them to nitrocellulose membranes. Western blot analyses were performed using various specific primary antibodies. The immunoreactive bands in the immunoblots were visualized with horseradish peroxidase-coupled goat anti-rabbit immunoglobulin using an enhanced chemiluminescence Western blotting system (ECL Plus, GE Healthcare Life Sciences, Pittsburgh, PA, USA). Nonspecific antigen sites were blocked with 10% bovine serum albumin in 1× Tris-buffered saline.

### Gelatin zymography

MMP-9 activity was measured with a gelatin-zymography kit (Primary Cell, Sapporo, Japan) according to the manufacturer’s instructions. Caov-3 cells were seeded in 6-well plates at 5 × 10^5^ cells/mL. Cells were cultured in serum-free DMEM for 24 hours and treated with PBS or 100 μM of Cisplatin or 100 nM of Gemcitabine or in combination for 12 hours at 37°C in a humidified, 5% CO_2_ atmosphere.

Thereafter, the cultured supernatants were collected and electrophoresed in gelatin at 15 mA constant for 90 minutes. After renaturing and developing the Gel, adding 40 mL of SimplyBlue Safestain and incubate for 1 hour at room temperature under gentle agitation. After staining, gels were scanned with a resolution of 300 dpi. And the respective band densitometry analyses were performed using the Image J software program.

### RNA extraction and semi-quantitative reverse transcription-polymerase chain reaction (RT-PCR)

Total RNA was obtained using the RNeasy Mini kit (Qiagen, Germantown, MD, USA) from cells starved for 24 hours then treated with PBS or 100 μM of Cisplatin for 24 hours with or without being pretreated with 100 nM of Gemcitabine for 12 hours. A total of 2 μg of total RNA was reverse-transcribed with Superscript II RNase H-reverse transcriptase (Invitrogen, Carlsbad, CA, USA) using random primers according to the manufacturer’s instructions. The cDNA (1 μl) was amplified using 0.1 μM of each primer, 1 U of Taq DNA polymerase (Roche Diagnostics, Mannheim, Germany), PCR buffer with 1.5 mM of MgCl_2_ and 0.25 mM of dNTPs in a 20-μl reaction volume using a PTC200 Thermal cycler (Bio-Rad Laboratories, Hercules, CA, USA). The amplification conditions were as follows: initial denaturation at 94°C for three minutes, followed by 28 cycles comprising denaturation at 94°C for 30 seconds, annealing at the optimized temperature for each set of primers for 30 seconds and extension at 72°C for 30 seconds. The final extension was carried out for five minutes at 72°C. The products were analyzed on 2.0% (w/v) agarose gels stained with 0.5 mg/ml ethidium bromide (Sigma-Aldrich) and visualized under an ultraviolet transilluminator. The product size was approximated using a 100-bp DNA ladder (Bangalore Genei, Bangalore, India). The negative control did not contain reverse transcriptase (RT) enzymes in the reaction mixture. The PCR primers for the Taqman/Probe Library assays were designed using the Probe Library Assay Design Center (Roche) as follows: VEGF-F:5′-AGGAGGAGGGCAGAATCATCA -3′, and reverse, VEGF-R: 5′-CTCGATTGGATGGCAGTAGCT -3′. Quantitative RT-PCR was performed on a LightCycler 2.0 (Roche Diagnostics, Indianapolis, IN), and the results were analyzed with the LightCycler Software program 4.05 (Roche Diagnostics) using a calibrator-normalized relative quantification approach. The relative gene expression quantification was normalized to the β-actin expression.

### *In vivo* growth inhibition assay

Female 6-week-old athymic nude mice (BALB/c Slc-nu/nu) were used for the tumor experiments. The mice had access to sterile food pellets and water ad libitum. The institutional guidelines for animal welfare and experimental conduct were followed. Caov-3 cells were suspended in PBS, after which 5 × 10^6^ cells were injected i.p. into each of the 20 female 6-week-old nude mice. Two weeks after the inoculation of Caov-3 cells, the athymic nude mice were randomly assigned to one of four groups treated with the following regimens for six weeks: (a) vehicle (PBS); (b) Cisplatin (5 mg/kg) once a week; (c) Gemcitabine (60 mg/kg) once a week; and (d) Cisplatin (5 mg/kg) once a week + Gemcitabine (60 mg/kg) once a week. The abdominal circumference was measured weekly. Eight weeks after the initiation of treatment, all mice were sacrificed.

### Apoptosis detection and immunohistochemistry

At sacrifice, the liver, bowels and uterus were excised, flushed with PBS and fixed in formalin overnight. Five-micron sections obtained from the liver, bowels and uterus of the mice were fixed in formalin and embedded in paraffin then prepared for the immunohistochemical analyses. The degree of apoptosis was analyzed with the ApopTag® Plus Peroxidase In Situ Apoptosis Kit (EMD Millipore Headquarters, USA), while the proliferation index was evaluated by staining for Ki67 and the microvessel density was evaluated by staining for CD31 antigens. The percentage of apoptotic cells and the MIB-1 index reflected the percentage of the total number of tumor cells with nuclear staining. In addition, the percentage of the vessel area was calculated as the ratio of the number of CD31-positive vessels in the tumor as an index of angiogenesis. All parameters were obtained in three different mice, and all values represent the mean ± SD of the results of three mice in each group.

### Statistics

The data represent the mean ± SD of three to five independent experiments. The statistical analysis was performed using Student’s *t*-test at a significance level of P < 0.05 to < 0.01. The symbol (*) indicates p < 0.05 and the symbol (**) indicates P < 0.01 compared with the control.

## Results

### Gemcitabine specifically enhances the Cisplatin-induced inhibition of cell viability

**Figure 1:**
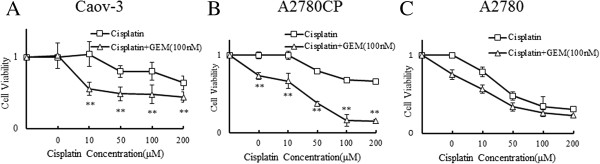
**Gemcitabine enhances the Cisplatin**-**induced inhibition of cell viability.** The anti-cancer drug sensitivity in the Caov-3 **(A)**, A2780CP **(B)** and A2780 **(C)** cells was examined using an MTS assay. The cells were treated with the indicated concentrations of Cisplatin with or without 100 nM of Gemcitabine (GEM). Twenty-four hours later, the cell viability was assessed using the MTS assay as described under “Materials and Methods”. Significant differences are indicated by asterisks. **, p < 0.01.

The sensitivity cell viability of Cisplatin in the Caov-3, A2780CP and A2780 cells was examined using an MTS assay. It was first confirmed that A2780 cells are sensitive and Caov-3 and A2780CP cells are resistant to Cisplatin, as previously reported [16]. As shown in Figure 1A,B and C, the viability of the Caov-3 and A2780CP cells, but not the A2780 cells, remained unaffected by increasing the concentration of Cisplatin to over 200 μM or that of Gemcitabine to over 1,000 nM. We examined whether the co-treatment with cisplatin and gemcitabine leads to synergistic inhibition of cell viability in the Caov-3 and A2780CP cells using a Mixlow method of Assessing Drug Synergism/Antagonism [22]. There was synergistic inhibition of cell viability in the Caov-3 (combination index (CI) = 0.74845) and A2780CP cells (CI = 0.49595) following the combined treatment with Cisplatin and Gemcitabine (Figure 1A and 1B, Additional file 1: Figure S1).

### Gemcitabine specifically enhances the Cisplatin-induced inhibition of cell invasion through Matrigel

**Figure 2:**
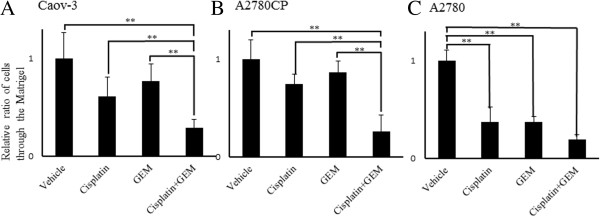
**Gemcitabine enhances the Cisplatin**-**induced inhibition of cell invasion.** Caov-3 **(A)**, A2780CP **(B)** and A2780 **(C)** cells were treated with or without 100 μM of Cisplatin for 24 hours in combination with 100 nM of Gemcitabine for 12 hours. The invasive potential of the cells was examined using an invasion assay, as described under “Experimental Procedures”. Significant differences from the vehicle group are indicated by asterisks. **, p < 0.01.

The sensitivity of the invasive activity of Cisplatin in the Caov-3, A2780CP and A2780 cells was examined using an assay of the invasion through Matrigel. We examined the effects of Cisplatin and Gemcitabine alone and in combination on the invasion of the Caov-3, A2780CP and A2780 cells through the Matrigel. Although neither Cisplatin nor Gemcitabine inhibited the invasion of the Caov-3 or A2780CP cells through the Matrigel, co-treatment with Cisplatin and Gemcitabine almost completely inhibited the invasion of both of these cell lines through the Matrigel (Figure 2A and 2B). In contrast, both Cisplatin and Gemcitabine almost completely inhibited the invasion of the A2780 cells through the Matrigel (Figure 2C).

### Cisplatin activates the Akt survival pathway in Cisplatin- resistant cell lines

**Figure 3:**
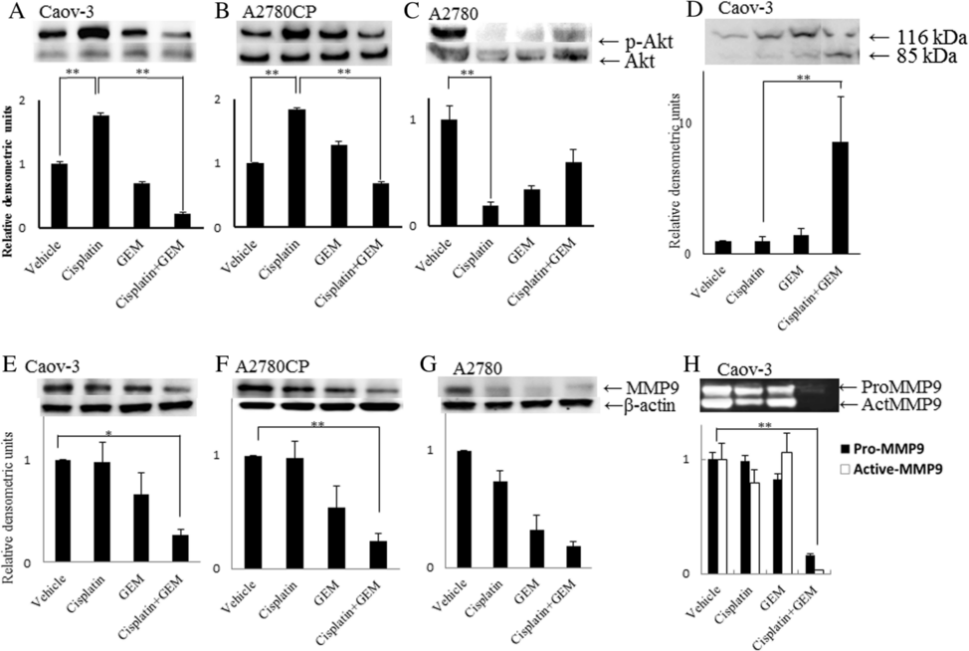
**Gemcitabine attenuates Cisplatin**-**induced survival Akt signals and enhances apoptosis in Cisplatin**-**resistant cell lines.** Caov-3 **(A)**, A2780CP **(B)** and A2780 **(C)** cells were treated with or without 100 μM of Cisplatin for 24 hours in combination with 100 nM of Gemcitabine for 12 hours. The cell lysates were subjected to SDS-PA GE, followed by a Western blot analysis with an anti-phospho-Akt (Ser473) antibody (top parts of **A**, **B** and **C**) and an anti-Akt antibody (bottom parts of **A**, **B** and **C**). The PARP proenzyme (116 kD) is indicated in the top parts, and the cleaved subunit (85 kD) is indicated in the bottom parts **(D)**. The levels of Cisplatin-induced MMP9 phosphorylation are indicated in the top parts of **E**, **F** and **G**, and the relative levels to the β-actin expression are indicated in the bottom parts of **E**, **F** and **G**. MMP-9 activity in Caov-3 cells was measured with a gelatin-zymography. The Pro-MMP9 is indicated in the top parts, and the Active-MMP9 is indicated in the bottom parts **(H)**. The respective band densitometry analyses were performed using the Image J software program. The values represent the mean ± SE of at least three independent experiments. Significant differences are indicated by asterisks.**p < 0.01, * p < 0.05.

We examined the Akt kinase activity following treatment with Cisplatin or Gemcitabine individually and in combination. We observed that Cisplatin induced Akt phosphorylation in both the Caov-3 and A2780CP cells, although there was no synergistic effect in the A2780 cells (Figure 3A, 3B and 3C). Gemcitabine had no effect on the levels of Akt phosphorylation. However, the combination of Cisplatin and Gemcitabine significantly inhibited the levels of Cisplatin-induced Akt phosphorylation, as shown in Figure 3A and 3B. Co-treatment with Cisplatin and Gemcitabine resulted in a 78% decrease in comparison to the Western blotting band intensities of phosphorylated Akt in the Caov-3 and A2780CP cells treated with Cisplatin alone.

We examined whether Gemcitabine affects the Akt activity induced by Cisplatin in the Caov-3 cells. PARP is a substrate of caspase-3 that is also cleaved to produce the 85 kDa apoptotic fragment [23]. Co-treatment with Cisplatin and Gemcitabine significantly induced the cleavage of PARP; however, Cisplatin did not induce cleavage of PARP in the Caov-3 cells (Figure 3D). These results suggest that Gemcitabine promotes apoptosis by suppressing the Akt kinas activity induced by Cisplatin in Caov-3 cells that are resistant to Cisplatin.

### Effects of the inhibition of the MMP9 activity in the Cisplatin-resistant cell lines induced by Gemcitabine

We examined the MMP9 activity following treatment with Cisplatin or Gemcitabine individually and in combination, as co-treatment with Cisplatin and Gemcitabine inhibited the invasion of both of these cell lines through the Matrigel. We observed that both Cisplatin and Gemcitabine had no effect on the levels of MMP9 activation in the Caov-3 cells compared with that observed in the Caov-3 cells treated without these agents. Gemcitabine had no effect on the levels of MMP9 activation. However, co-treatment with Cisplatin and Gemcitabine resulted in a 78% decrease in comparison to the Western blotting band intensities of phosphorylated MMP9 in the Caov-3 cells treated with Cisplatin alone, as shown in Figure 3E. We also examined the MMP-9 activity using a zymographic assay. We observed that neither cisplatin nor gemcitabine had a significant effect on the levels of MMP9 activation in the Caov-3 cells compared with that observed in the Caov-3 cells treated without these agents. However, co-treatment with cisplatin and gemcitabine resulted in a 97% decrease in the zymographic assay band intensities of active MMP9 compared to the Caov-3 cells treated with cisplatin alone, as shown in Figure 3H.

### Gemcitabine blocks the vascular endothelial growth factor expression induced by Cisplatin

**Figure 4:**
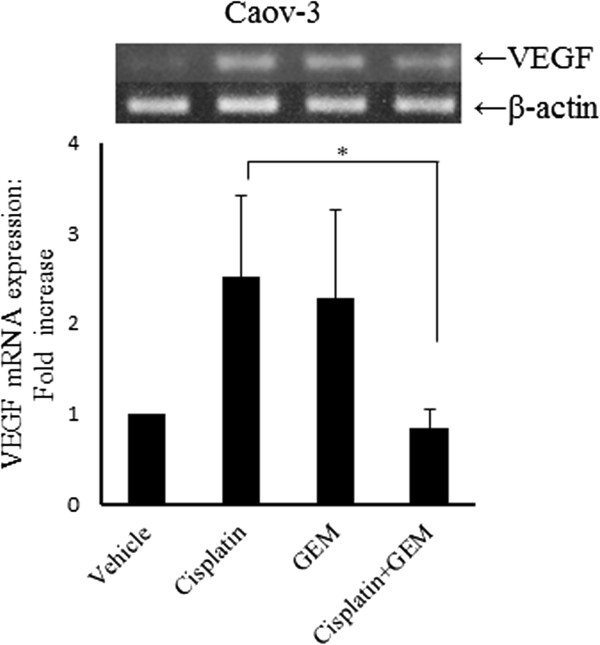
**Gemcitabine significantly inhibits the Cisplatin**-**induced VEGF gene expression in Caov**-**3 cells.** The cells were treated with or without 100 μM of Cisplatin for 24 hours in the presence or absence of 100 nM of Gemcitabine for 12 hours. Total RNA was isolated and reverse transcribed, and the resulting cDNA was used for PCR to detect the VEGF gene (upper panel). The level of β-actin was detected as an internal control (lower panel).* p < 0.05.

We examined the VEGF expression in the Caov-3 cells treated with vehicle, Cisplatin alone, Gemcitabine alone or the combination of Cisplatin and Gemcitabine using an RT-PCR analysis (Figure 4). The combination of Cisplatin and Gemcitabine significantly decreased the expression of the VEGF gene compared with that achieved by Cisplatin alone. These results indicate that combination therapy consisting of Cisplatin and Gemcitabine inhibits not only the Akt activity, but also the VEGF expression induced by Cisplatin treatment (Additional file 2: Figure S2).

### Effects of Gemcitabine on the Cisplatin-induced inhibition of intra-abdominal dissemination of ovarian cancer

**Figure 5:**
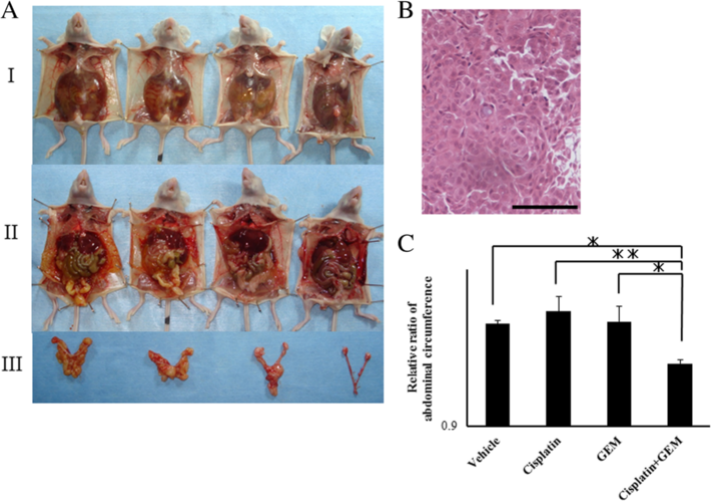
**Gemcitabine enhances the cisplatin**-**induced inhibition of intra**-**abdominal dissemination.** Athymic nude mice were inoculated i.p. with Caov-3 cells. Two weeks after the inoculation of Caov-3 cells, as described in the Materials and Methods section, the athymic mice were randomly assigned to one of four groups treated with different regimens for six weeks. **(A)** Physical appearance of representative mice. The combination of Cisplatin and Gemcitabine reduced the production of ascites (I) and intra-abdominal dissemination (II and III). **(B)** Magnified views of the intra-abdominal dissemination pattern in the vehicle mice and histological findings (×200 magnification) of hematoxylin and eosin staining of parietal peritoneal dissemination in the athymic nude mice. **(C)** Relative ratios of the abdominal circumferences in each group. The combination of Cisplatin and Gemcitabine significantly decreased the mean abdominal circumference six weeks after the initiation of treatment. Significant differences are indicated by asterisks. **p < 0.01, *p < 0.05.

Peritoneal dissemination is the primary route of progression in human ovarian cancer, and the amount of ascites and disseminated tumor burden correlates with the patient prognosis in humans [24]. We therefore examined the effects of treatment with Cisplatin and Gemcitabine alone and in combination on the control of intra-abdominal ovarian cancer dissemination, ascites formation and tumor growth in order to assess whether combination therapy increases the therapeutic efficacy of each agent. Athymic nude mice were inoculated i.p. with Caov-3 cells, as described in the Materials and Methods Section. The appearance of the mice is shown in Figure 5A-I. Intra-abdominal dissemination was clearly detected in the athymic nude mice inoculated i.p. with Caov-3 cells followed by treatment with PBS (Figure 5A-II and 5A-III). The combination of Cisplatin and Gemcitabine further enhanced the inhibitory effects on the production of ascites and intra-abdominal dissemination (Figure 5A-II, 5A-III). After performing a histological examination (Figure 5B), the abdominal tumors were found to be papillary adenocarcinomas, consistent with the characteristics of Caov-3 cells. The mean abdominal circumference six weeks after the initiation of treatment in the mice treated with combination therapy consisting of Cisplatin and Gemcitabine was significantly lower than that observed in the mice treated with PBS or Cisplatin alone (Figure 5C), suggesting that the production of ascites was inhibited by treatment with Gemcitabine. The disseminated tumor volume six weeks after the initiation of treatment in the mice treated with combination therapy consisting of Cisplatin and Gemcitabine was also significantly lower than that observed in the mice treated with PBS or Cisplatin alone (Figure 5D, Additional file 3: Figure S3).

### Gemcitabine inhibits the angiogenic activity induced by Cisplatin and induces the apoptotic activity in the intra-abdominal disseminated ovarian cancer model

**Figure 6:**
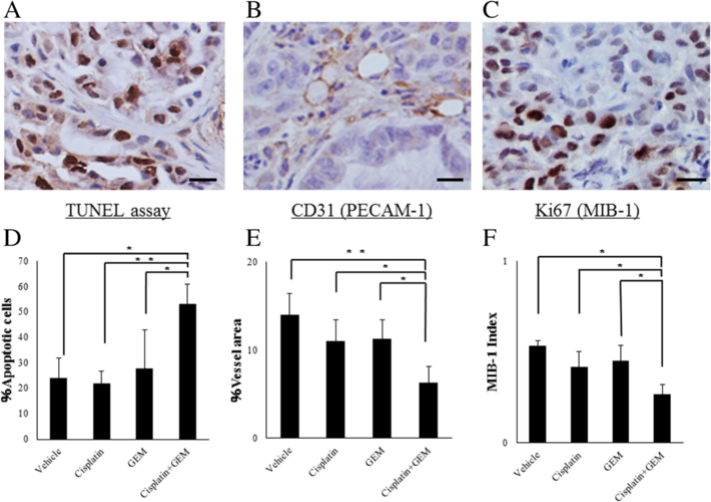
**Analysis of apoptosis and angiogenesis in the tumors growing intra**-**abdominally in the athymic nude mice model.** Five-micron sections obtained from the liver, bowels and uterus of the athymic nude mice were fixed in formalin and embedded in paraffin then prepared for the immunohistochemical analyses. The combination of Cisplatin and Gemcitabine significantly induced apoptosis **(A)**, inhibited the angiogenic activity **(B)** and depressed the MIB-1 index **(C)**. Significant differences are indicated by asterisks. **p < 0.01, *p < 0.05.

We next examined whether Gemcitabine induces the apoptotic activity and inhibits the proliferative and angiogenetic activity *in vivo*. Figure 6A shows the apoptotic activity measured using a TUNEL assay. The number of apoptotic cells was significantly increased upon combined treatment with Cisplatin and Gemcitabine compared to that observed in the mice treated with the vehicle, Cisplatin alone or Gemcitabine alone. The intratumoral microvessel density and Ki67 index significantly decreased upon combined treatment with Cisplatin and Gemcitabine compared to those observed in the mice treated with the vehicle, Cisplatin alone or Gemcitabine alone (Figure 6B, C). These results indicate that Cisplatin and Gemcitabine combination therapy significantly induces apoptosis and inhibits the proliferative and angiogenic activity.

## Discussion

In this study, we demonstrated synergism between the effects of Gemcitabine and Cisplatin in several ovarian cancer cell lines. The most pronounced effects were observed in the Cisplatin-resistant cell lines. The management of platinum-resistant ovarian cancer remains unsatisfactory. Resistance to Cisplatin is a multifactorial phenomenon, the elements of which may be placed in three general categories: (a) reduced intracellular accumulation of Cisplatin, (b) elevated levels of glutathione and metallothionein, and (c) increased tolerance or repair of DNA damage [25-27]. Because Cisplatin acts by forming intrastrand and interstrand DNA cross-links and DNA protein cross-links, thus resulting in DNA damage, overcoming these lesions with heightened repair mechanisms is an important factor for Cisplatin resistance [28]. We previously reported that the PI3K/Akt cascade plays a role in Cisplatin resistance [12-14]. It has been demonstrated that Cisplatin resistance is primarily due to the reduction of DNA damage and the evasion of apoptosis, the latter of which includes the loss of damage recognition and activation of the PI3K/Akt pathway [29,30]. Although it is well known that Gemcitabine is one of the most active drugs in patients with platinum-resistant ovarian carcinoma, the mechanisms underlying these phenomena have not yet been characterized. In previous studies, Peters G.J., et al. showed that the combination of Cisplatin and Gemcitabine produces synergistic effects in platinum-resistant ovarian cancer cells [28]. Moufarij M.A., et al. and Ledermann J.A., et al. demonstrated the inhibitory effects of Gemcitabine on the repair of Cisplatin-induced intrastrand adduction and interstand cross-linking in platinum-resistant ovarian cancer cells [31,32]. However, these authors did not indicate either the mechanisms or the signal cascades underlying the synergistic effects of such combination treatment with Cisplatin and Gemcitabine in platinum-resistant ovarian cancer cells. In this study, we found that combination treatment with Cisplatin and Gemcitabine significantly inhibits the level of the Cisplatin-induced Akt activity in Cisplatin-resistant cell lines (Caov-3 and A2780CP cells). We clarified that Gemcitabine exerts its cytotoxic effects by interfering with the antiapoptotic machinery and significantly enhancing PARP cleavage. Moreover, we found that combination treatment with Cisplatin and Gemcitabine significantly inhibits both the levels of invasive activity and the Cisplatin-induced MMP9 activity in Caov-3 and A2780CP cells; however, these effects are not achieved with treatment with either Cisplatin or Gemcitabine alone. We found that Cisplatin induced the VEGF expression in the Cisplatin-resistant cell lines. Combined treatment with Cisplatin and Gemcitabine significantly inhibits the Cisplatin-induced VEGF expression in platinum-resistant ovarian cancer cells, although no such effects are observed after treatment with either Cisplatin or Gemcitabine alone. Moreover, we found that combined treatment consisting of Cisplatin and Gemcitabine significantly inhibits intra-abdominal tumor cell dissemination and ascites production compared to that observed following treatment with Cisplatin or Gemcitabine alone. Whether these phenomena are represented by other anticancer agents is unclear. It has been reported that Topotecan, a topoisomerase-1 inhibitor, inhibits the Akt and VEGF cascade in platinum-resistant ovarian cancers [16]. However, 5-fluorouracil (5-FU), which exhibits similar *in vitro* findings to the thymidylate synthase inhibitor, has no effect on the synergistic inhibition of cell viability in Caov-3 cells after combined treatment with Cisplatin and 5-FU, and does not inhibit the Cisplatin-induced Akt activity in Caov-3 cells (Additional file 4: Figure S4A, S4B). We found that Gemcitabine is a most effective molecular targeting agent with the ability to suppress the Akt kinase activity, the ability to induce cellular apoptosis and an anti-angiogenic activity in platinum-resistant ovarian cancer cell lines.

In the current clinical trial, the response rate to treatment with Gemcitabine and Cisplatin ranged from 16% to 64% in the Cisplatin-resistant ovarian carcinomas with measurable disease, although the frequency of grade 3 and 4 neutrophil toxicity ranged from 20% to 81.5% and the platelet toxicity ranged from 36% to 96.5% [19,20,33-35]. It is said that one must take into consideration that the use of less toxic and single agent drugs may be worthwhile in a treatment process that may span many years. However, previous clinical studies have shown that Gemcitabine and Cisplatin can be used to treat patients who have developed platinum-resistance and failed to respond to other second-line therapies over time, using a combination therapy that may result in an appropriate response [20]. In the present study, we were unable to show whether other factors, such as a reduced accumulation of Cisplatin or elevated levels of glutathione and metallothionein, affect the resistance of Cisplatin-resistant ovarian cancer. Such knowledge may be helpful for developing future strategies to more effectively circumvent the multifactorial mechanisms of platinum resistance. We believe that our data provide scientific justification for both previous and future trials of combination treatment with Gemcitabine and Cisplatin in patients with platinum-resistant ovarian cancer.

In conclusion, we herein demonstrated that Gemcitabine inhibits the Akt kinase activity and angiogenetic activity following treatment with Cisplatin in platinum-resistant ovarian cancer cells. These results provide a rationale for using Gemcitabine in clinical regimens containing molecular targeting agents against platinum-resistant ovarian cancers.

## Abbreviations

PI3K-Akt: Phosphatidylinositol 3-kinase-Akt; RT-PCR: Reverse transcription polymerase chain reaction; PARP: Poly (ADP-ribose) polymerase; MMP9: Matrix metalloproteinase 9; VEGF: Vascular endothelial growth factor; 5-fluorouracil: 5-FU; MTS assay: 3-(4,5-dimethylthiazol-2-yl)-5-(3-carboxymethoxyphenyl)-2-(4-sulfophenyl)-2H-tetrazolium assay; CI: Combination index.

## Competing interests

The authors declare that they have no competing interests.

## Authors’ contributions

HK, AT, KA and TT performed the MTS assay, invasion assay, Western blot analysis, zymographic assay and statistical analysis. HT, SF and HS conducted the cancer cell cultures and the extraction of mRNA for RT-PCR and protein for the Western blot analysis, performed part of the gene expression experiments and cultured the cells. HK and ST provided the athymic nude mice, performed the tumor cell injections and collected the peritoneal disseminated tumors. YT participated in the conception and design of the study and drafted the manuscript. HK, AT and YT participated in designing the study. YoT and MK evaluated the histology of the tumor samples and the immunohistochemical staining. MO contributed methodological knowledge and participated in designing the study. All authors read and approved the final manuscript.

## Supplementary Material

Additional file 1: Figure S1 Gemcitabine sensitivity in Caov-3 (A), A2780CP (B) and A2780 (C) cells. The cells were treated with Gemcitabine at various concentrations for 24 hours. The number of viable cells was assessed using an MTS assay, as described in the Materials and Methods section. **p < 0.01. , *p < 0.05. Click here for file

Additional file 2: Figure S2 Effect of combination treatment with Cisplatin and Gemcitabine on the VEGF mRNA expression in A2780CP (A) and A2780 (B) cells. The cells were treated with various combinations of 100 nM of Gemcitabine and 200 μM of Cisplatin for six hours. Total RNA was isolated and reverse transcribed, and the resulting cDNA was used in PCR for the semi-quantification of the VEGF mRNA expression relative to that of β-actin. The values represent the mean ± S.E.M. of at least three separate experiments. Significant differences are indicated by asterisks. **p < 0.01. Click here for file

Additional file 3: Figure S3 Effects of Cisplatin and Gemcitabine on tumor growth *in vivo*. Athymic nude mice were inoculated i.p. with A2780CP cells. Two week after inoculation, as described in the Materials and Methods section, the athymic mice were inoculated i.p. with A2780CP cells. (A) Physical appearance of representative mice. The combination treatment with Cisplatin and Gemcitabine reduced tumor production. (B) Magnified views of the tumor in the Vehicle mouse and the histological findings (×200 magnification) of hematoxylin and eosin staining. (C) Relative ratio of the abdominal circumference (I) and tumor weight (II) in each group. The combination therapy with Cisplatin and Gemcitabine significantly decreased the mean abdominal circumference and tumor weight six weeks after the initiation of treatment. Significant differences are indicated by asterisks. **p < 0.01, *p < 0.05. Click here for file

Additional file 4: Figure S4 Effects of 5-FU on cell viability and Akt phosphorylation in Caov-3. (A) The cells were treated with Cisplatin at various concentrations with (□) or without (×) 100 nM of 5-FU for 24 hours. The number of viable cells was assessed using an MTS assay, as described in the Materials and Methods section. (B) The cells were treated with various combinations of 100 nM of Gemcitabine and 200 μM of Cisplatin for 10 minutes. The cell lysates were subjected to Western blotting for phosphor-Akt (upper panel) and Akt (lower panel), with the density of the control bands arbitrarily set at 1.0. The values represent the mean ± S.E.M. of at least three separate experiments. Significant differences are indicated by asterisks. **p < 0.01. Click here for file
