# Supplementary figures and images for: Gemcitabine as a molecular targeting agent that blocks the Akt cascade in platinum-resistant ovarian cancer
Source: J Ovarian Res. 2014 Apr 9;7:38. doi: 10.1186/1757-2215-7-38 (PMC4234938; doi:10.1186/1757-2215-7-38)

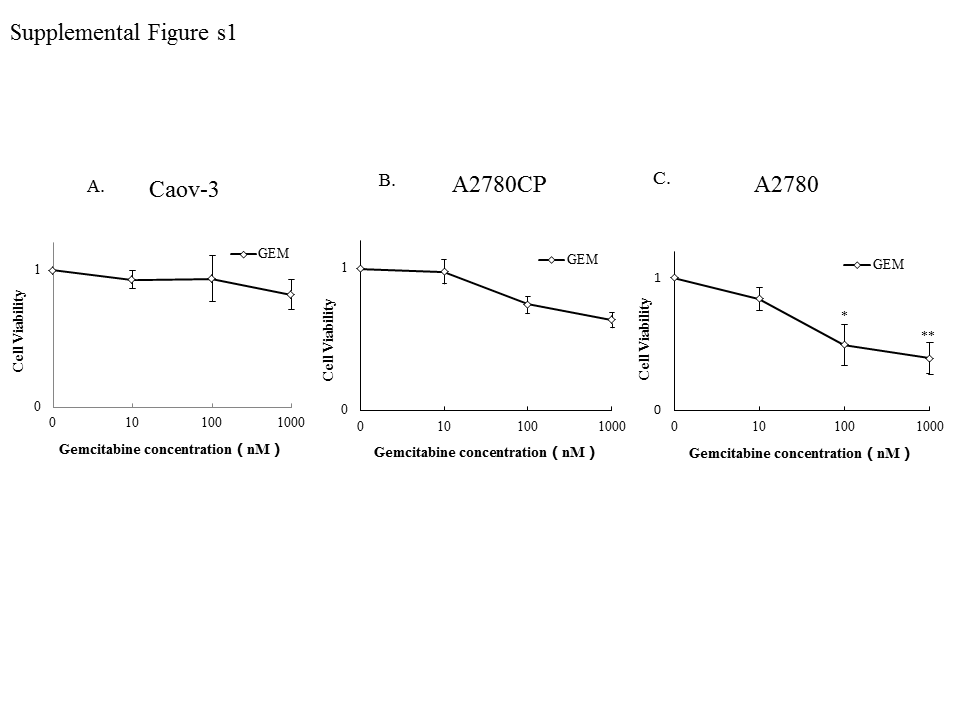

Supplement: Additional file 1: Figure S1 — Gemcitabine sensitivity in Caov-3 (A), A2780CP (B) and A2780 (C) cells. The cells were treated with Gemcitabine at various concentrations for 24 hours. The number of viable cells was assessed using an MTS assay, as described in the Materials and Methods section. **p < 0.01. , *p < 0.05. [file 1757-2215-7-38-S1.TIFF]

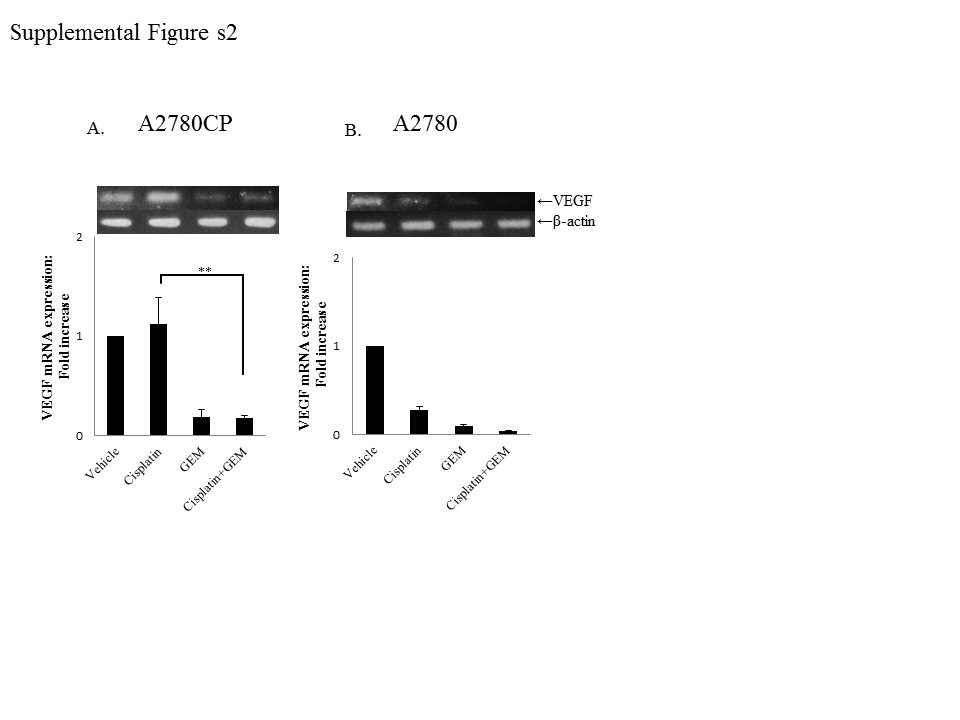

Supplement: Additional file 2: Figure S2 — Effect of combination treatment with Cisplatin and Gemcitabine on the VEGF mRNA expression in A2780CP (A) and A2780 (B) cells. The cells were treated with various combinations of 100 nM of Gemcitabine and 200 μM of Cisplatin for six hours. Total RNA was isolated and reverse transcribed, and the resulting cDNA was used in PCR for the semi-quantification of the VEGF mRNA expression relative to that of β-actin. The values represent the mean ± S.E.M. of at least three separate experiments. Significant differences are indicated by asterisks. **p < 0.01. [file 1757-2215-7-38-S2.TIFF]

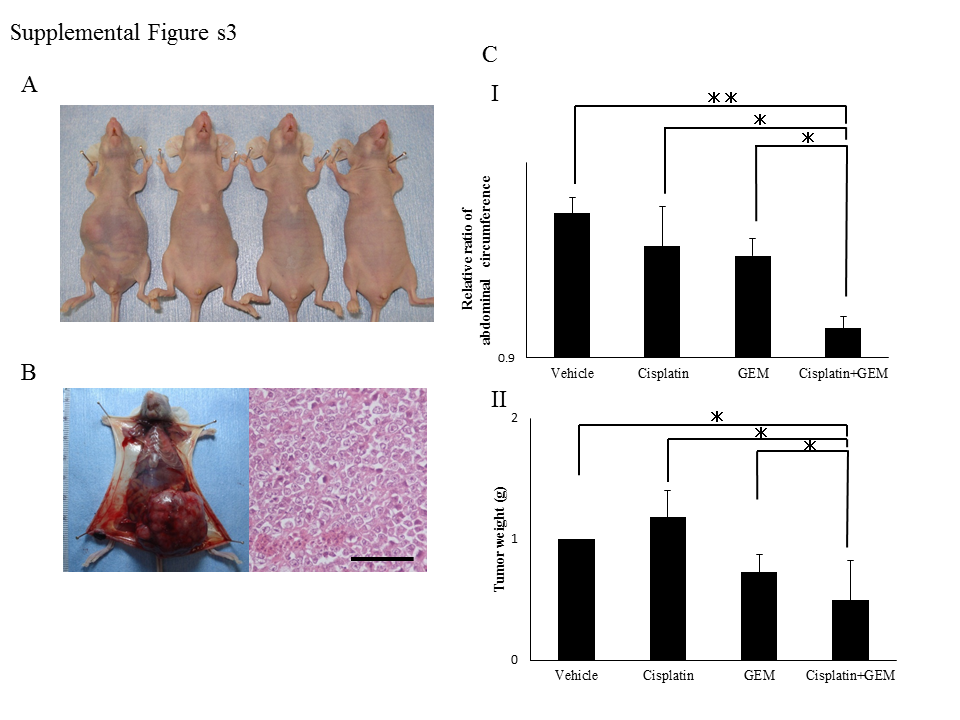

Supplement: Additional file 3: Figure S3 — Effects of Cisplatin and Gemcitabine on tumor growth in vivo. Athymic nude mice were inoculated i.p. with A2780CP cells. Two week after inoculation, as described in the Materials and Methods section, the athymic mice were inoculated i.p. with A2780CP cells. (A) Physical appearance of representative mice. The combination treatment with Cisplatin and Gemcitabine reduced tumor production. (B) Magnified views of the tumor in the Vehicle mouse and the histological findings (×200 magnification) of hematoxylin and eosin staining. (C) Relative ratio of the abdominal circumference (I) and tumor weight (II) in each group. The combination therapy with Cisplatin and Gemcitabine significantly decreased the mean abdominal circumference and tumor weight six weeks after the initiation of treatment. Significant differences are indicated by asterisks. **p < 0.01, *p < 0.05. [file 1757-2215-7-38-S3.TIFF]

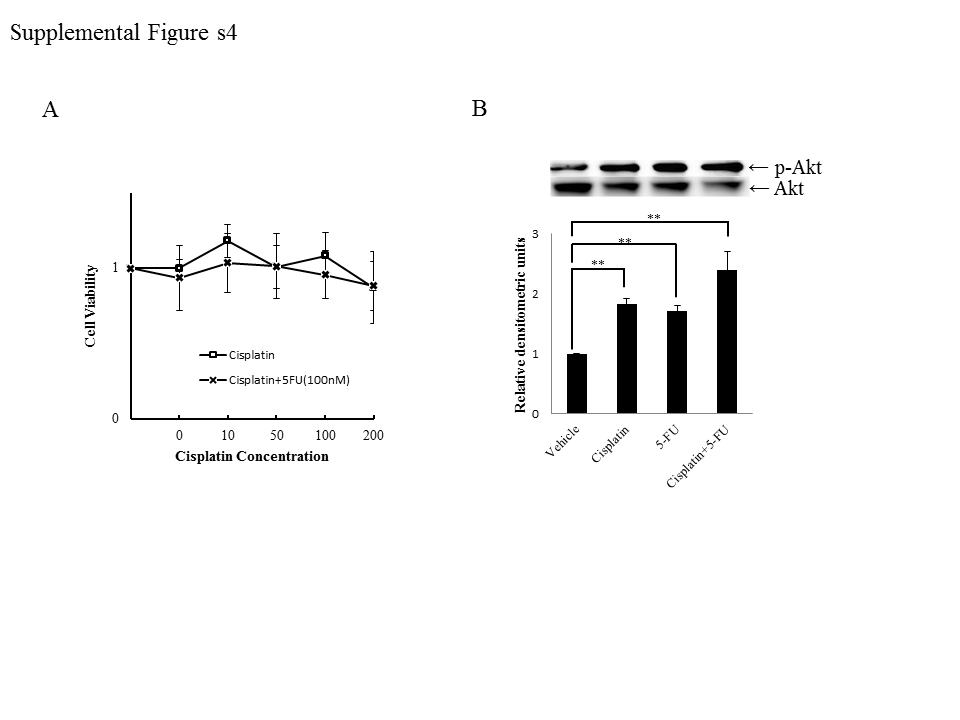

Supplement: Additional file 4: Figure S4 — Effects of 5-FU on cell viability and Akt phosphorylation in Caov-3. (A) The cells were treated with Cisplatin at various concentrations with (□) or without (×) 100 nM of 5-FU for 24 hours. The number of viable cells was assessed using an MTS assay, as described in the Materials and Methods section. (B) The cells were treated with various combinations of 100 nM of Gemcitabine and 200 μM of Cisplatin for 10 minutes. The cell lysates were subjected to Western blotting for phosphor-Akt (upper panel) and Akt (lower panel), with the density of the control bands arbitrarily set at 1.0. The values represent the mean ± S.E.M. of at least three separate experiments. Significant differences are indicated by asterisks. **p < 0.01. [file 1757-2215-7-38-S4.TIFF]
